# Human Auditory Detection and Discrimination Measured with the Pupil Dilation Response

**DOI:** 10.1007/s10162-019-00739-x

**Published:** 2019-12-02

**Authors:** Avinash D. S. Bala, Elizabeth A. Whitchurch, Terry T. Takahashi

**Affiliations:** 1 Institute of Neuroscience, University of Oregon, 228 Huestis Hall, Eugene, OR 97403-1254 USA; 2 Facilities Management, Humboldt State University, 1 Harpst Street, Arcata, CA 95521 USA

**Keywords:** orienting, audiometry, auditory detection, auditory discrimination, involuntary response, orienting response, orienting reflex, habituation, recovery, oddball, oddball paradigm, pupil dilation, pupillometry, human

## Abstract

In the standard Hughson-Westlake hearing tests (Carhart and Jerger 1959), patient responses like a button press, raised hand, or verbal response are used to assess detection of brief test signals such as tones of varying pitch and level. Because of its reliance on voluntary responses, Hughson-Westlake audiometry is not suitable for patients who cannot follow instructions reliably, such as pre-lingual infants (Northern and Downs 2002). As an alternative approach, we explored the use of the pupillary dilation response (PDR), a short-latency component of the orienting response evoked by novel stimuli, as an indicator of sound detection. The pupils of 31 adult participants (median age 24 years) were monitored with an infrared video camera *during* a standard hearing test in which they indicated by button press whether or not they heard narrowband noises centered at 1, 2, 4, and 8 kHz. Tests were conducted in a quiet, carpeted office. Pupil size was summed over the first 1750 ms after stimulus delivery, excluding later dilations linked to expenditure of cognitive effort (Kahneman and Beatty 1966; Kahneman et al. 1969). The PDR yielded thresholds comparable to the standard test at all center frequencies tested, suggesting that the PDR is as sensitive as traditional methods of assessing detection. We also tested the effects of repeating a stimulus on the habituation of the PDR. Results showed that habituation can be minimized by operating at near-threshold stimulus levels. At sound levels well above threshold, the PDR habituated but could be recovered by changing the frequency or sound level, suggesting that the PDR can also be used to test stimulus discrimination. Given these features, the PDR may be useful as an audiometric tool or as a means of assessing auditory discrimination in those who cannot produce a reliable voluntary response.

## INTRODUCTION

Human hearing is typically evaluated using the voluntary reports of patients or research subjects. In some settings, whether clinical or research, voluntary reports are infeasible either because the patient or subject cannot be expected to follow the instructions reliably, or because instructions are incompatible with the research question or procedure. In such cases, clinicians and researchers may use physiological tests where appropriate. We propose here an alternative technique for assessing auditory detection, based on an autonomic response, the acoustically evoked pupillary dilation response (PDR), which requires no voluntary reports on the part of subject or patient.

The PDR is a component of the orienting response evoked by novel stimuli and is part of a suite of “covert” responses such as changes in skin conductance and changes in heart rate, which accompany overt orienting such as the turning of the head, eyes, and ears in the direction of the novel stimulus (Liberman 1958; Sokolov 1963; van Olst 1971). The PDR’s latency is about 0.25 s in humans, which is significantly shorter than that of the tonic dilation that builds up over several seconds as cognitive effort is exerted (Kahneman and Beatty 1966; Kahneman et al. 1969; Johnson 1971; Zekveld et al. 2014). Further, while the PDR, like other components of the OR, is characterized by rapid, stimulus-specific habituation and recovery in response to novel stimuli, responses evoked by cognitive load are largely indifferent to stimulus repetition. Finally, use of the PDR to assess detection and discrimination was widespread in earlier decades (1950–1970), while most current studies tracking pupil diameter focus on cognition (for example, see Liberman 1958; Sokolov 1958; Geer 1966; Levine and Whitney 1970; Shakhar et al. 1975; Granholm et al. 1997; Koelewijn et al. 2012, 2014; Unsworth and Robison 2015).

In the barn owl (*Tyto alba*), an animal model of spatial hearing, PDR was used to measure detection thresholds, minimal audible angles, and frequency discrimination (Bala and Takahashi 2000; Bala et al. 2003; Spitzer et al. 2003). Importantly, the birds in these studies were not trained, other than having been acclimated to remaining still with their head fixed. The success of the PDR in probing the owl’s auditory performance suggested that it may also be useful in evaluating various aspects of human hearing, ultimately, under conditions in which voluntary tasks are infeasible.

The goal of the present study was to describe the general features of the human PDR in a population of adults (median age = 24 years) with no self-reported hearing loss. Below, we first describe the shape, size, and timing characteristics of the PDR. We then show that the sensitivity of the PDR for estimation of thresholds was comparable to, if not more sensitive than, those obtained during a Hughson-Westlake-like procedure conducted while the pupil was monitored. Finally, we describe the habituation of the PDR and its recovery by changes in frequency and amplitude, affirming that the response we studied is the novelty-evoked orienting response, not the longer-term dilation evoked by tasks that involve cognitive effort and memory load. Characterization of the dilation response we tracked as an OR has implications for its potential use, and for comparing such data acquired using the PDR to data from other components of the OR, such as the galvanic skin response.

## METHODS

### Subjects

This study was conducted under a protocol approved by the Institutional Review Board of the University of Oregon.

Results are based on the performances of 12 male and 18 female volunteers who replied to ads posted around the University of Oregon (UO, Eugene, OR) campus. Their median age was 24 years (22 years without a 63-year male subject). Subjects had no self-reported hearing loss, and applicants were excluded only if they were unable to see a small dot on a computer monitor 95 cm away or maintain gaze at the dot without blinking for 5 s.

### Testing Environment

**Fig. 1:**
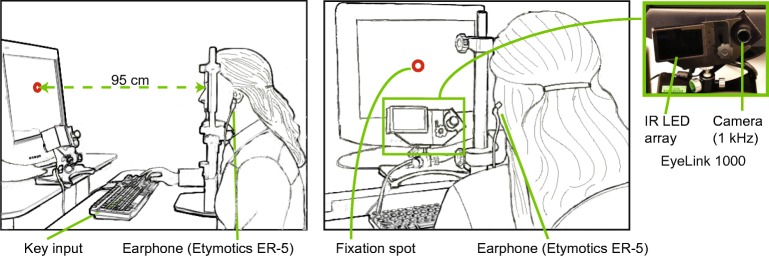
PDR apparatus. The subject, wearing insert earphones, was seated facing a computer monitor (95 cm), keyboard, IR LED array, and IR sensitive camera. The subject’s head was stabilized by chin and forehead rests. The monitor displayed a fixation point (*red circle*) which turned into a question mark, prompting the subject to press one key if a sound was detected and a different key if no sound was detected. The subject’s left pupil was monitored by the camera which was interfaced to Eyelink software that provided pupillary areas at a rate of 1000 samples/s

Subjects were tested in a laboratory room in Straub Hall at the University of Oregon (Eugene, OR). The laboratory (width = 3.7 m; length = 4.8 m; height = 2.75 m) had no special sound treatment other than a carpet and Seltex ceiling. The ambient sound level near the subject’s head ranged between 23.2 and 23.8 dBA (dB SPL_A_), measured using a 1/2-in. microphone (Brüel and Kjær 4176) and sound-level meter (Brüel and Kjær 2625). The ambient light level ranged from 90 to 128 lx (6.5 to 7 EV; measured using a Sekonic L-188 light meter). Pupil size was tracked using a commercially available eye-tracking system (EyeLink 1000, SR Research Ltd., Mississauga, ON, Canada) consisting of a bank of infrared light-emitting diodes (IR LEDs) and an IR sensitive camera, positioned on the desk immediately below the monitor and thus below the subject’s gaze. As shown in Fig. 1, the subject sat at a desk facing the “subject monitor” and keyboard, while the experimenter controlled the experiment from a different computer in the same room, but remained seated out of the subject’s view. The subject’s head was maintained at a distance of 95 cm from the monitor with chin and forehead rests.

### Stimulus Synthesis and Presentation

Stimuli consisted of 100 ms narrowband noises (gammatones) with 5 ms trapezoidal on- and off-ramps. Narrowband noises were created in Matlab by first generating bursts of random noise, which were then passed through gammatone filters. Waveforms were stored offline as uncompressed *wav* files, converted to analog signals (Lexicon Omega digital audio interface; 44,100 samples/s; 32-bit resolution), and presented diotically over insert earphones (Etymotics ER5). Headphone output was calibrated by presenting 3 kHz tones in the free field—a center frequency not used in test sounds in this study—and using miniature Knowles microphones (model EM4046) to measure in-ear sound level, which was then compared to sound levels measured in the free field to assess the SPL of each stimulus. Stimulus details specific to Experiments 1 and 2 are presented below in “METHODS” *(Experiment 1 design; Experiment 2 design*) along with descriptions of the individual experiments in “RESULTS.”

### PDR Measurement and Quantification

The EyeLink 1000 is a commercial product designed for tracking eye position, but it also provides a measure of pupil size, which is related to the number of pixels included in the image of the pupil (SR Research, private communication). Since subjects used a head and chin rest that was fixed to the edge of the table, the subject distance remained at constant 95 cm. The relationship between the “pupil size” measure provided by the Eyelink device and actual pupil diameter was consistent across sessions and subjects, allowing for direct comparisons of stimulus-evoked events. We tracked the left eye for all subjects, at a 1000-Hz sampling rate.

When pupil size is tracked across time (e.g., Fig. 3a), an upward deflection of the size trace corresponds to a dilation, and a downward deflection to a constriction. The area under the trace was integrated between 0.25 and 2 s after sound onset, yielding an estimate of PDR response magnitude.

Responses obtained from trials repeated with the same stimulus were averaged. The pupil size at the time of trial onset was set to zero. No other filtering or signal processing was performed.

### Experiment 1 Design—Simultaneous PDR and Voluntary-Response Audiometry

**Fig. 2:**
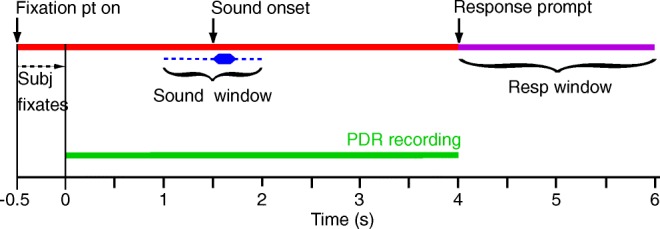
Sequence of events during a trial. Upon fixating on a small circle on the computer monitor, the subject’s pupil was video imaged (*green*) and the pupil sizes were readout by Eyelink software. A sound (blue) was presented 1.5 s (+/− 0.5 s) after the onset of video imaging. The sound’s delivery was jittered to prevent pupillary responses in anticipation of a sound. The circle turned into a question mark, which prompted the subject to respond within 2 s (*purple*) if they detected a sound

To compare the sensitivity of the PDR to that of a voluntary response, we tracked a subject’s pupil size while (s)he performed the *delayed response task* (DRT), a modified version of the standard hearing test—the Hughson-Westlake procedure (Carhart and Jerger 1959). The DRT is schematically illustrated in Fig. 2. Subjects were asked to direct their gaze at a small red circle in the center of the LCD monitor that was 95 cm away for approximately 5 s, and were instructed to do so without blinking if possible (“*Fixation pt on*”). The red circle subtended 40 pixels in diameter (< 0.1 °), with a line thickness of 20 pixels, leaving the central 20 pixels black. When a subject looked directly at the fixation circle, the experimenter initiated the trial and tracking of pupil size (*green line*) commenced. Stimuli were presented on average at 1.5 s after trial initiation, but the trial-by-trial stimulus onset was randomly jittered by ± 0.5 s (“*Sound window*”) to prevent subjects from reacting in anticipation of a sound. Pupil size was tracked for an additional 2 s after sound onset, after which the tracking stopped, and the fixation circle on the monitor changed to a question mark, thereby indicating the start of a 2-s response window during which subjects indicated via key-press whether or not they heard a sound. Thus, overall trial duration varied between 5 and 6 s, which included a 1- to 2-s pre-sound period, a 2-s post-sound onset recording period, and a 2-s button-press response window. This approach allowed us to compare the PDR with the voluntary response in the same subject on a trial-by-trial basis.

In Experiment 1, stimuli were gammatone-filtered noises (See “Stimulus Synthesis and Presentation”) with center frequencies ranging from 0.5 to 8 kHz in one-octave steps, each of which was presented at four different levels of 13, 23, 33, and 43 dBA. These levels were chosen by first estimating thresholds (23 subjects) using a 3-kHz gammatone probe using the DRT. Using a probe at a different frequency allowed us to minimize the effects of pre-exposure. Since every subject could detect at least one of the four probe levels, the range of stimulus levels (13–43 dB) was left unchanged. Most subjects were able to detect the 3-kHz gammatone at 33 dBA, but not at 23 dBA. Sounds were presented in batches of 30 trials, comprising 20 sound trials (five different frequencies at four levels each) and 10 interspersed catch (no-sound) trials.

During catch trials, which comprised 1/3 of all trials, we presented an analog conversion of a *wav* file containing a string of zeros. The catch-trial “stimuli” were generated, stored, converted to analog and otherwise handled exactly like a sound, serving as a control for any conversion artefacts that may have arisen during the analog presentation of digital stimuli or during amplification. We used a block design to order stimuli, in which each block contained one iteration of each frequency and level combination arranged in pseudo-random order. However, we made sure that no sound at a higher level was followed by a lower level sound at the same center frequency. Subjects completed 5 blocks each, resulting in sessions of 150 trials, including 100 sound trials and 50 catch trials. Aborted trials, if any, were repeated at the end of the block. Sessions lasted between 10 and 30 min, depending on the number of trials that were aborted if subjects blinked, pressed a key early, or their gaze wandered by more than 5 °. On average, there were 23 aborted trials per session. Subjects who reported for multiple sessions showed improvement with practice, with second session averages of 12.27 aborts, compared to 23.90 in the first session (*t* test, *p* = 0.12). The majority of aborts (93 %) were due to blinks during the PDR recording period, rather than a loss of fixation.

Subjects were free to blink during the 2-s response period or between trials. To minimize aborted trials, subjects were instructed to look away from the fixation point if they needed to blink, and only re-direct their gaze at the target when they had blinked for as many times or for as long as they needed. Most subjects blinked 1–2 times between trials. Trials were initiated after they had redirected their gaze at the fixation point and the direction of gaze met the criterion for stability.

The consistency of the technique was examined by retesting individual subjects (*n* = 21 subjects) from 1 to 11 times (median = 3 times) with intervals ranging from 2 to 146 days (median = 7 days).

### Experiment 2 Design—Habituation and Recovery of the PDR

#### Habituation

In Experiment 2, we first characterized the habituation of the PDR. Twelve subjects listened to the same suprathreshold gammatone (52 dBA; center frequency = 1 kHz) presented 150 times at a presentation rate of one stimulus per *≈* 8 s. In a separate series of experiments, with 12 different subjects, 100 sounds were presented in blocks of 20, with a 4-min pause separating each block. Within a block, stimuli were presented at a rate of one stimulus per *≈* 8 s. Subjects completed 5 blocks, yielding at least 3, and in most cases, 5 repetitions of each frequency-level combination (see Fig. 10). As in Experiment 1, we asked subjects to press a key after each sound to report the detection of the repeated sound. At the SPL employed, sounds were expected to be audible, and all subjects indicated detection during all trials. Meanwhile, the subject’s pupil was monitored as described above.

Habituated responses are known to recover spontaneously after a break from exposure to the habituating stimulus. To test for spontaneous recovery, we asked subjects to return at their earliest convenience. Subjects returned 0–14 days later (median interval of 7 days). For subjects who returned on the same day (0 days), the interval between habituation sessions was 23–38 min with a median of 24 min.

#### Recovery

After determining the characteristics of PDR habituation, we examined whether the response would recover upon presentation of “oddball” gammatones that differed from the habituating stimulus (1 kHz; 52 dBA) in *either* amplitude or center frequency. Table 1 shows the parameters of habituating and recovery sounds used in the two recovery experiments. Sessions comprised 155 trials, in 149 of which the standard 1-kHz tone was presented. During the first 48 trials, only the habituating sound was presented to allow for habituation. After the first presentation of the oddball in the 50th trial, the oddball tone was presented every 21 ± 2 trials, for a total of 6 oddball presentations per session.

**Table 1:** Habituation and recovery parameters. Habituation was induced by the repeated presentation of a 52-dB, 1-kHz gammatone. We tested for recovery by changing either the amplitude (to 38 dB) or gammatone frequency (to 2 kHz) in separate experiments

	Habituating sounds	Recovery (oddball) sounds
Experiment: SPL oddball	1 kHz; 52 dBA	1 kHz; 38 dBA
Experiment: Frequency oddball	1 kHz; 52 dBA	2 kHz; 52 dBA

### Statistical Analysis

Data from Experiment 1 (Audiometry) were analyzed by accumulating null (catch trial) and test (sound trial) distributions. The test and null distributions were compared using receiver-operating characteristic (ROC) analysis (Egan 1975; Britten et al. 1992). ROC analysis yields a function, the area under which is equivalent to the quantity “proportion correct” [*p(C)*]. If the null and test distributions are indistinguishable, then *p(C)* = 0.5, which corresponds to chance performance. The further apart the test and null distributions are, the larger the area under the ROC curve and higher the *p(C)*. The *p(C)* is computed for parameters of a test stimulus, in our case the SPL, and by plotting *p(C)* against the parameter, we derive a psychometric function (see Fig. 6a, solid lines). The parameter value at which *p(C)* first exceeded 0.75 (halfway between floor and saturation) was arbitrarily chosen as the threshold.

Key-press responses in the DRT revealed that our pool of young, normal hearing adult subjects could either reliably detect sounds, or could not detect them at all: responses at each sound level were either almost all hits or almost all misses with very few false alarms (Gutschalk et al. 2008). As a consequence, these data could not be quantified as *p(C)*, and are instead represented as proportion hits (% hits, which is equivalent to % yes). In this case, psychometric functions (dashed lines, Fig. 6a), ranged between 0 (0 % hits or 100 % miss) and 100 (100 % hits or 0 % miss). In order to be conservative, so that PDR performance was not over-estimated, the parameter value at which % hits first exceeded 50 (halfway between floor and saturation) was arbitrarily chosen as the threshold. Note, however, that changing the arbitrary threshold—for example, to 75 %—does not affect any of our conclusions about the relative sensitivity of the PDR, compared to key-press responses.

Data from Experiment 2, which tested the habituation and recovery of the PDR, were first normalized by converting the PDR responses to *z-*scores, using the mean and variance of the habituating (catch) trials. The first 20 habituating trials were excluded for the purpose of computing *z-*scores, ensuring that the first few trials—when habituation was largely incomplete—did not influence the test for recovery. Recovery during test trials would result in *z-*scores greater than 0. Normalized data were pooled across subjects, and catch trial responses were compared to test responses using an unpaired *t* test (two-tailed, *p =* 0.05). Individual trial data from Experiment 1 could also be converted into *z-*scores to allow for pooling across sessions for an individual subject, and across subjects (Figs. 5, 10), while population responses were expressed as *p(C)*, computed by ROC analysis as described above.

## RESULTS

### General Properties of the Human PDR

**Fig. 3:**
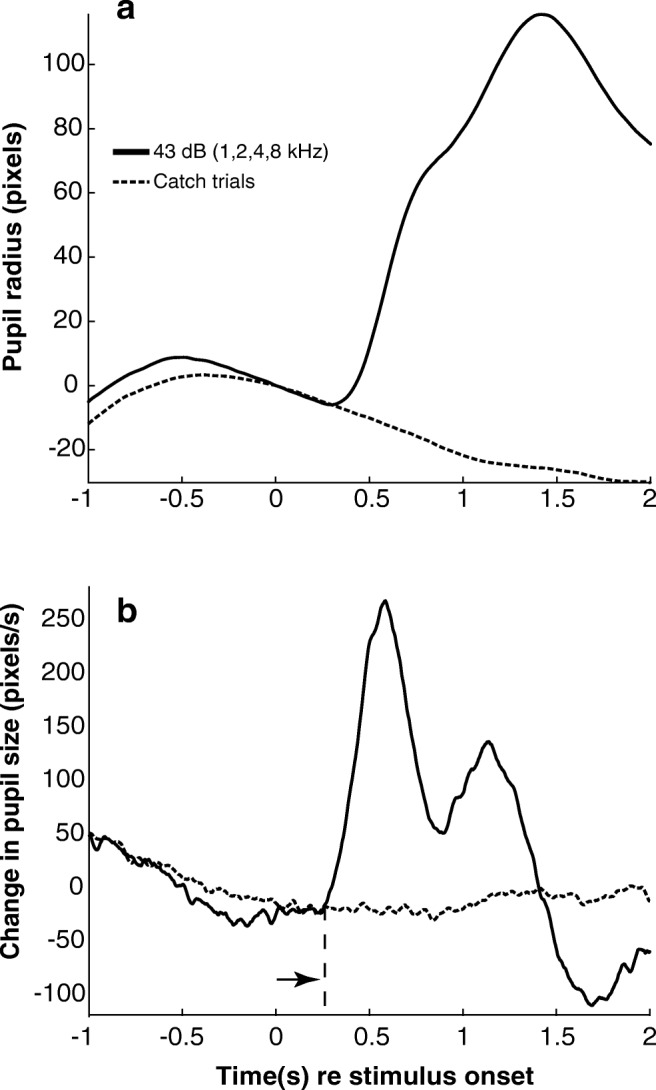
PDR time course and latency. **a***Solid curve:* PDR averaged across 1673 test trials presented at the highest sound level used for four center frequencies (43 dBA; 1, 2, 4, 8 kHz center frequencies), drawn from 84 sessions across 21 subjects. Stimulus onset is at 0 s on the abscissa. *Dashed curve:* PDR averaged across 4191 catch trials from all 84 sessions across 21 subjects. **b** First derivative of the PDR trace from (**a**) shows the average latency of the PDR (*arrow*) and reveals the two-phase dynamics of pupil dilation in response to sound presentation

Figure 3a shows the average PDR elicited by sounds at 43 dBA at all center frequencies (1, 2, 4, 8 kHz gammatones). Positive numbers and an upward deflection indicate a dilation, while negative numbers and a downward deflection indicate a constriction. The solid line shows that the sound-elicited PDR has a fast onset, followed by a later, slower rise, resulting in a “shoulder” at about 0.75 s post stimulus onset (0 s). The PDR peaks at about 1.4 s. By comparison, intervals with no sound presentation (dashed line) show no dilation at the corresponding times. The latency of the averaged PDR was determined by computing the first derivative of the trace shown as a solid line in Fig. 3a. The derivative, shown in Fig. 3b, rises rapidly at about 0.25 s (arrow near bottom, Fig. 3b) after sound onset (0 s). Thus, at 43 dB, the latency of the average PDR is about 250 ms. PDR properties were best assessed using averaged dilations, since size during individual trials varies due to the hippus, the periodic and small oscillation in pupil size (McLaren et al. 1992).

**Fig. 4:**
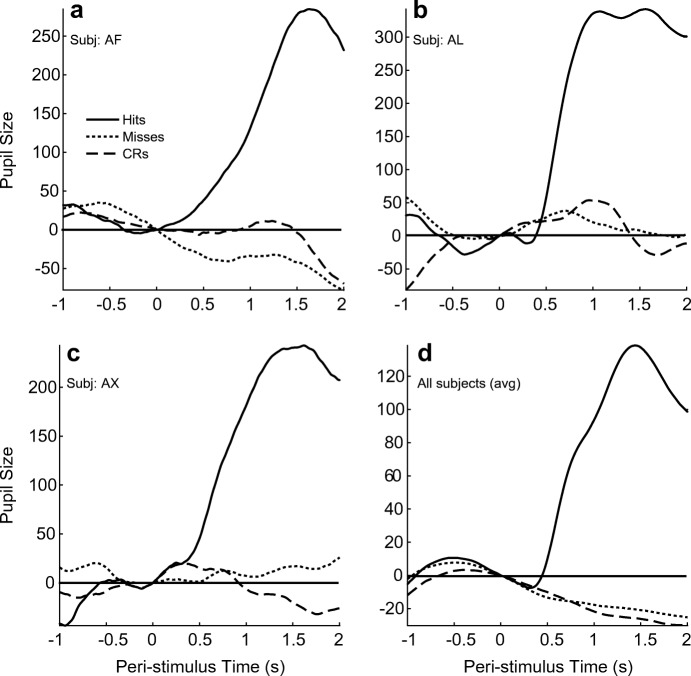
Detectable sounds elicit a larger PDR. **a–c** Data from 3 typical subjects (*AF*, *AL*, *AX*). Pupil size traces were pooled across 100 sound trials and 50 catch trial presentations in a single session for each subject. Sound trials were pooled across all center frequencies tested (1, 2, 4, and 8 kHz) at the 4 standard sounds levels. The PDRs were later classified according to subjects’ button presses in the DRT conducted while the pupil was monitored: *Hit* (sound was present, subject pressed “yes” button), *Miss* (sound was present; subject pressed “no” button), and *Correct Rejection* (CR; subject pressed the “no” button during a catch trial). A dilation is seen only when the subjects reported a sound (solid line), while sounds that were undetectable yielded a pupil size trace (dashed line) that was similar to responses during catch trials (dotted line). Each trace is the average of all trials of its type in the session (Hits, Misses, CRs respectively: **a**: 24, 61, 43; **b**: 30, 68, 50; **c**: 16, 82, 50). Dilation responses were compared using unpaired *t* tests (two-tailed; *p* = 0.05): responses during Hits were significantly different from that during CRs, while responses during CRs and Miss trials were statistically indistinguishable. **d** PDR averaged across all trials and subjects. As with the individual subjects (**a**–**c**), a large dilation was observed only when a sound was detected. Traces are averages of 2233 Hits, 6141 Misses, and 4191 CRs, pooled across all 23 subjects and 85 sessions. Dilation responses in the pooled data were easily discriminable when comparing *Hits* to *Misses* (*p* < 10^–97^), while responses between *Miss* and *CR* trials were indiscriminable (*p* = 0.32)

Figure 4a–c illustrates the properties of the sound-elicited dilation response from three typical subjects (*AF, AL, AX*). Note that the prominent dilation is present only during trials during which a sound was presented, following which the subject indicated that the sound had been detected by their pressing the “yes” button. By contrast, no dilation is seen during trials without sounds (catch trials), following which the subject indicated a failure to detect any stimulus by pressing the “no” button (*Correct Rejections* (*CRs*)). Furthermore, trials during which a sound was presented, but resulted in the “no” button being pressed (*Misses*), also showed no evidence of a dilation. Note that pupil size during *Hits* and *CR* trials is significantly different, while dilation during *CRs* and *Misses* is statistically indiscriminable (unpaired *t* test; *p* = 0.05). The same pattern is seen when pupil size is averaged across all subjects (Fig. 4d). Here, the number of trials that were averaged are in the thousands, instead of the tens of trials in subplots a–c. In the pooled data too, a large dilation is seen only in trials following which the subjects pressed the “yes” button (*Hits*), while a dilation is not seen during trials that either had no sound (*CRs*) or where sound was presented but was not detected (*Misses*). The dilation response during trials categorized as *Hits* is significantly different from response during trials categorized as Misses or CRs (unpaired *t* test; *p* < 10^−97^), while the dilation response during the latter two is indistinguishable (unpaired *t* test; *p* = 0.59). The response window was delayed relative to the stimulus onset, to separate an acoustically evoked PDR and a possible dilation that might result from the intent to push the response key (Richer et al. 1983). Also, by ensuring that subjects pressed a key whether or not they heard a sound, we controlled for dilations solely due to preparation for executing a motor task (Hakerem and Sutton 1966). Thus, the absence of dilation during “no” trials shows that the dilation response was not elicited by the intent to push a button or the motor act thereof. Thus, it is reasonable to conclude that the dilation response we tracked was elicited by detected sounds.

### Experiment 1—PDR Magnitude Increases with Increase in SPL

In the first experiment, we assessed the relationship between SPL and PDR magnitude in 21 subjects who contributed a total of 85 sessions (median of 3 sessions per subject). Pupil size was tracked during DRT, while we presented a gammatone in random order at 5 different center frequencies (0.5–8 kHz in octave steps) and 4 SPLs (13, 23, 33, 43 dBA). About a third of the trials were catch trials, during which no sound was presented. The 0.5-kHz stimulus, however, elicited no detectable PDR, nor did the subjects report (by button press) having detected the 500-Hz tone, even at the highest SPL tested. The likely reason for this observation is the inefficiency of the small headphone transducers in the ER5 headphones to effectively produce a 500-Hz tone at a level comparable to higher frequency sounds. Therefore, we did not further analyze the 500-Hz data.

**Fig. 5:**
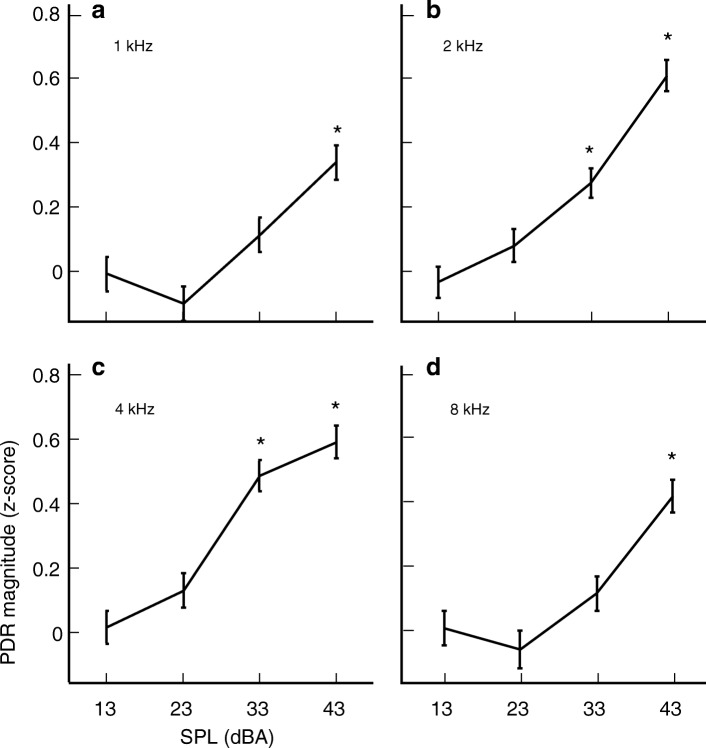
PDR magnitude vs SPL. Average magnitude of the PDR vs SPL across all 85 sessions from 21 subjects for center frequencies of 1 (**a**), 2 (**b**), 4 (**c**), and 8 kHz (**d**). Each point represents the average PDR sizes expressed as a *z-*score relative to PDRs obtained in catch trials (*n* = 4191). Each data point is the average of > 400 trials—trial numbers for each frequency at sound levels of 13, 23, 33, and 43 dB, respectively, are as follows: **a**: 421, 419, 419, and 414 trials; **b**: 412, 421, 417, and 419 trials; **c**: 415, 413, 413, and 415 trials; **d**: 408, 411, 414, and 415 trials. *Asterisks* indicate PDRs that are statistically greater (*p* < 0.05) in magnitude than those obtained in catch trials. Error bars represent the SEM

Figure 5 plots the *z-*scores of the PDRs against SPL for the four test frequencies, in a manner similar to a psychometric function. For each test frequency, the size of the PDR increases, nearly monotonically, as SPL increases. The asterisks in each panel indicate the SPL at which the PDR magnitude was significantly larger (*p* < 0.05) than those observed in catch trials. Thus, for the 2- and 4-kHz gammatones, the PDR was significantly larger at 33 and 43 dBA, while at 1 and 8 kHz, only the 43-dBA stimuli evoked a significant PDR suggesting that the subjects were more sensitive to the 2- and 4-kHz gammatones.

### Comparison of PDR and DRT

**Fig. 6:**
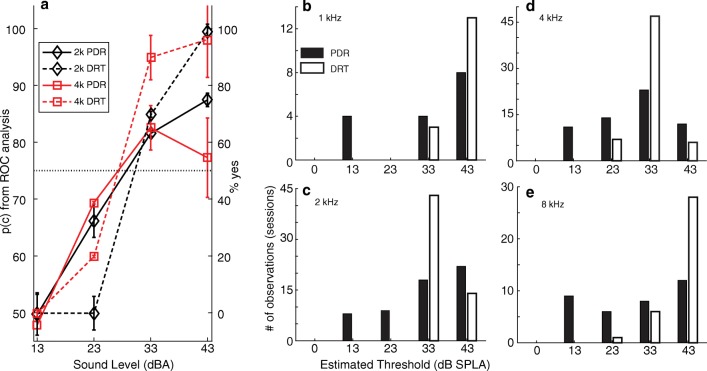
Comparison of PDR and DRT in individual subjects. **a** Average psychometric functions derived from PDR (pupil size; solid lines) and DRT (button press; dashed lines) data. Example curves are shown for 2 kHz (black lines and diamonds) and 4 kHz (red lines and squares). Bars are SEM. **b–e** Distribution of thresholds obtained with PDR (black bars) and DRT (unfilled bars) for 1, 2, 4, and 8 kHz test stimuli. PDR and DRT data were simultaneously gathered in each subject. Overall PDR thresholds are lower than DRT thresholds but PDR variances are wider. Details of the results are given in Tables 1 and 2. Thresholds extracted from psychometric functions for individual sessions—the averages of which are shown in **a**—was used to construct histograms in subfigures **b**–**e**. Thus, thresholds extracted from pooled data represented by black lines (**a**) was used to construct histograms in **c**, and thresholds from pooled data represented by red lines (**a**) was used to construct the histograms in **d**

The PDR was monitored in each subject while they performed the DRT, in which they pressed separate buttons depending on whether or not they detected the test gammatones with center frequencies of 1, 2, 4, or 8 kHz. In this section, we first compare the simultaneously obtained PDR and DRT thresholds in individual subjects to get a preliminary idea of their relative sensitivity. We then compare their variability. Psychometric curves constructed using PDR (*p(c)*) and DRT (*% yes*) for two example frequencies—2 and 4 kHz—are shown in Fig. 6a. These curves, constructed using *pooled* data, show that PDR and DRT sensitivities are similar.

Thresholds derived from psychometric functions for *individual* sessions are compared in Fig. 6, which plots the number of sessions (ordinate) in which a particular threshold (abscissa) was observed for 1-, 2-, 4-, and 8-kHz test frequencies (b–e). As shown, the PDR (black bars) yields lower thresholds than the DRT (unfilled bars) measured concomitantly at each frequency, although there is more variability for the PDR.

Table 2 compares the thresholds obtained using the PDR and DRT. As shown (Table 2, column "Diff"), the PDR-derived thresholds are from ≈ 3 to 11 dB lower than those derived from the DRT. This difference was statistically significant for all frequency bands tested (paired *t* test; see Table 2 for probabilities). Our data thus suggest that in each subject, the thresholds estimated with the PDR are comparable to, if not lower, than those obtained with the DRT, a voluntary task.

**Table 2:** Comparison of PDR and DRT thresholds. The means and standard deviations of each individual subject’s thresholds obtained with the PDR and DRT in the same trial are shown. From left to right, the columns represent the following: center frequency (*kHz*); the number of trials conducted with the PDR and DRT (*# observ.*); the average thresholds across subjects obtained with the PDR *(PDR mean*); the *sd* for the PDR (*PDR sd*); the average thresholds across subjects obtained with the DRT *(Avg DRT Th*); *sd* for the DRT (*DRT sd*); the difference in thresholds obtained by PDR and DRT (*Diff*); and the *p* values for the difference between the PDR and DRT results. Negative differences indicate that DRT thresholds were higher

Ctr freq (kHz)	# observ.	PDR mean	PDR *sd*	DRT mean	DRT *sd*	Diff	*t* test
1	16	33	13	41	4	− 8	*p* < 0.05
2	57	32	11	35	4	− 3	*p* < 0.05
4	60	29	10	33	5	− 4	*p* < 0.005
8	35	30	12	41	5	− 11	*p* < 0.001

How consistent are the thresholds estimated by PDR and DRT within an individual tested multiple times? As a measure of within-subject repeatability, we computed the standard deviations (*sd*) of the thresholds obtained in an individual’s multiple sessions, in those that participated in two or more sessions. Results are summarized in Table 3 which shows, for each center frequency, the average *sd*s across subjects for PDR and DRT (Table 3, *avg sd PDR; avg sd DRT*). The PDR results were more variable at each frequency, i.e., less repeatable from session to session, than the DRT results.

**Table 3:** Comparison of PDR and DRT variability. The repeatability of the PDR and DRT results was compared by examining the session-to-session variability of the two methods. From left to right, the columns represent the following: center frequencies (*kHz*); the average *sd* for PDR results; number of sessions from which the averages were computed from the PDR (*# of observations PDR*); the averaged *sd* for DRT results, the number of sessions from which the averages were computed from the DRT (*# of observations DRT*), the difference in the PDR and DRT *sd*s (*Diff*; positive numbers indicate that the *sd* obtained with the PDR is larger than that obtained with the DRT.); and the results of an *f* test

Ctr freq (kHz)	Avg *sd* PDR	# observ PDR	Avg *sd* DRT	# observ	Diff	*f* test
1	10	11	3	9	7	*p* < 0.05
2	9	17	2	21	7	*p* < 0.005
4	10	18	3	22	7	*p* < 0.01
8	11	12	1	16	10	*p* < 0.005

To summarize, our data suggest that the PDR can be at least as sensitive as the DRT, but is more variable from session to session. The possible reasons for these differences are addressed in “DISCUSSION.”

### Experiment 2—Habituation and Recovery of the PDR

We examined whether the sound-elicited dilation we tracked habituated upon repeated stimulation by a given stimulus, and whether the habituated response could be recovered by a novel stimulus in a stimulus-specific manner: both of these are characteristic of components of the orienting response. Habituation was tested by presenting a single stimulus repeatedly. Recovery was tested by first habituating the response, and then presenting a stimulus that was sufficiently different so as to be novel. A habituating-recovery paradigm can potentially be exploited as a reporting tool to examine sensory discrimination by habituating the PDR with one set of stimulus parameters and testing for recovery by altering one of the parameters, an approach we have previously used to determine auditory discrimination thresholds in barn owls (Bala and Takahashi 2000; Bala et al. 2003, 2007; Spitzer et al. 2003).

#### Habituation

To explore the characteristics of habituation, we monitored the PDR in 12 subjects while they performed the DRT, pressing a button whenever they heard a sound. Each subject was tested one to six times (average = 3 times) resulting in 36 total sessions. Since the SPL was always significantly above the subjects’ thresholds, they pushed the yes-key on all trials. The effects of changes in frequency or SPL are presented separately below (*Recovery—SPL; Recovery—Frequency*).

During a session, a listener heard 48 repetitions of the same narrowband noise (“habituating” stimulus; 1 kHz center frequency, 52 dBA) before either its SPL or center frequency was altered. The altered, or “oddball,” stimulus was presented six times, interspersed among the trials with the habituating stimulus. Below, we first describe the features of habituation after which we turn to the effects of altering the SPL or center frequency.

**Fig. 7:**
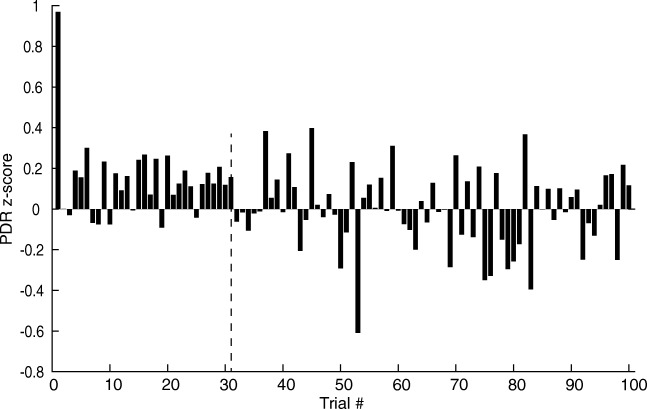
PDR habituation. Mean PDR magnitude (*ordinate*) vs trial number (*abscissa*). Data are based on 36 sessions (100 trials/session) in 12 subjects. After a large dilation on the first stimulus presentation, the dilations rapidly decline, but a small dilation remains for another 32 trials. The later trials (> 32; *dashed vertical line*) have larger variances that fluctuate around the mean PDR size (*z-score = 0*) computed across all trials. Comparison of the first 30 and last 70 trials show that the first 30 trials are significantly more positive than the last 70 trials (*p < 0.001; t test: Two-Sample Assuming Unequal Variances*)

Figure 7 shows the normalized PDR magnitude (*z-*scores; 36 sessions, 12 subjects) measured during the presentation of the habituating stimuli, averaged across all sessions. The first presentation evokes a large PDR, visible on the far left of Fig. 7, which quickly drops upon the presentation of the second and subsequent stimuli. This drop was typical of individual listeners although its size differed between listeners.

Figure 7 also shows that despite the rapid drop between the first and second trials, the PDR remains largely positive (i.e., dilated) up to about the 30th trial (*dashed vertical line*), suggesting that the PDR has not yet completely habituated. Between the 30th and 100th trials, there are both negative and positive *z-*scores and the scatter increases, indicating that stimuli no longer evoke consistent dilations. We compared the mean responses (*z-*scores) obtained in trials 1–30 against those obtained in trials 31–100 using a *t* test, which showed that the responses obtained during the first 30 trials were significantly larger (i.e., more positive) than those obtained during the remaining 70 trials (*p < 0.001*; 1-tailed, assuming unequal variances). This analysis suggests that despite the rapid initial drop, habituation of the PDR remains incomplete over the first ≈ 30 trials. Below, the average PDR obtained in trials 31–100 was used to represent the habituated state of the PDR.

#### Recovery—SPL

**Fig. 8:**
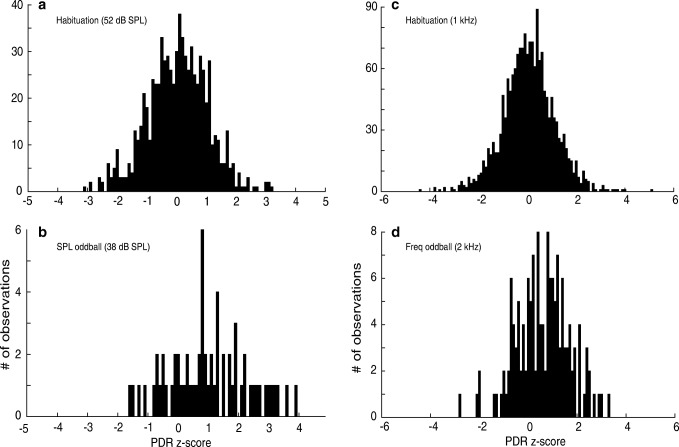
Distribution of PDR magnitudes during habituation and recovery. **a** Distribution of PDR magnitudes during habituating trials. (*mean = − 0.048; sd = 0.51; n = 561 trials*). **b** Distribution of PDR magnitudes when the SPL is changed. *(“SPL oddball”; mean = 0.96; sd = 0.35; n = 63 trials*). The PDRs evoked by oddball stimuli (*2 kHz gammatone; 38 dBA*) are statistically significantly larger (*p < 0.005; t test: Two-Sample Assuming Unequal Variances*) than those in the habituating trials (*1 kHz gammatone; 52 dBA*). **c** Distribution of PDR magnitudes during habituating trials (*12 subjects, mean = 0.002; sd = 0.23; n = 1750 trials*) and frequency oddball trials. **d** Distribution of PDR magnitudes when the center frequency is changed (“Freq oddball”; *mean = 0.54; sd = 0.30; n = 146 trials*). The PDRs evoked by oddball stimuli (*2 kHz gammatone; 52 dBA*) are statistically significantly larger (*p < 0.01; t test: Two-Sample Assuming Unequal Variances*) than those from habituating trials (*1 kHz gammatone; 52 dBA*)

**Fig. 9:**
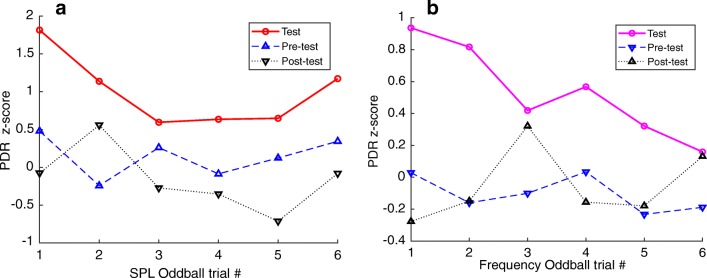
Recovered PDR to Oddball stimuli. Recovery elicited by oddball sounds at different SPL (**a**) and center frequency (**b**). Habituating stimuli, consisted of the 1-kHz gammatone at 52 dBA stimulus in both **a** and **b**, while the oddballs differed in sound level (**a**: 38 vs 52 dB), or in center frequency (**b**: 2 vs 1 kHz), as detailed in Table 1. The first oddball was presented during trial 49, and succeeding oddballs were separated by 21 ± 3 habituating stimuli. The PDR habituated to the lower-SPL oddball stimuli (**a**: red line and circles) or different center frequency (**b**: magenta line and circles) over the course of 6 trials. The magnitude of the PDR to the first presentation of the oddball in both cases was similar to the magnitude of the PDR elicited by the first habituating sound in each session (*t* test, *p* = 0.05). However, responses to subsequent presentations of the oddball stimulus were smaller, indicating habituation to the oddball stimuli. By the third repetition, responses to oddball stimuli were significantly smaller than responses to the first habituating trial of each session. While the response to oddball stimuli decreased over the course of the session, it still remained larger than responses to habituating stimuli (blue and black lines in **a** and **b**). Further, the recovery was specific to the oddball stimuli: the magnitude of habituating trials flanking the oddballs remained low. The PDR magnitude of habituating trials immediately preceding (black dotted line, upright triangles) and following (blue dashed line, inverted triangles) shows that responses to habituating stimuli were unaffected by the larger response to the oddball. Data in **a** are based on 63 oddball trials from 6 subjects in 11 sessions. (sessions with subjects *AE* and *AH* had 5 and 4 oddball trials respectively; the rest of the subjects had 6 oddballs). Data in **b** are based on 146 oddball trials from 12 subjects (one session for subject AQ had 5 oddball trials, while one for subject AE had only 3)

How do habituated subjects respond to presentations of novel stimuli? Our prior studies in barn owl suggest that the habituated PDR should recover when an oddball stimulus was presented. First, we tested the effect of *reducing* sound level from 52 to 38 dB, while leaving the center frequency at 1 kHz. Figure 8 shows the distributions of PDR magnitudes obtained in habituating (a; *n* = 561) and in oddball trials (b; *n* = 63; 7 subjects, 11 sessions). A comparison shows that the oddball trials yield significantly larger PDRs than the habituating trials (*p < 0.005*; *two-sample t test assuming unequal variances*). Earlier, we demonstrated that the PDR size scales with the SPL (Fig. 5). The observation that the PDR recovered after *decreasing* the SPL argues that the subject detected *a change* in the amplitude, and that the increased PDR magnitude in the context of habituation and recovery was unrelated to sound level, but to the apparent novelty of the oddball. As a novel sound, even quieter sounds (red line and circles; Fig. 9a) elicit a PDR that is larger than flanking habituating sounds that were 13 dB louder (dashed blue line and upright triangles, and dotted black lines and inverted triangles; Fig. 9a). This shows that the size of the PDR is context-dependent, and is not hard-wired to the level of the stimulus.

Figure 9, which plots the *z-*scores of PDRs evoked by oddball stimuli against trial number in 7 subjects (red line and circles), suggests that responses to the oddball stimulus itself habituate over time, despite the large number of intervening habituating trials, and the longer interval between oddball presentations.

#### Recovery—Frequency

Next, we tested for recovery of the habituated PDR by changing center frequency. The 1-kHz narrowband noise was repeated, after which the center frequency increased to 2 kHz during oddball trials. The amplitude was held constant at 52 dBA (Table 1).

Figure 8 shows the distribution of *z-*scores from all habituating (c) and oddball trials (d) in 12 subjects (25 sessions). A statistical comparison shows that the PDR evoked by the change in center frequency is larger, statistically, than those evoked in the habituating trials (*t test; p < 0.01*; 1-tailed, assuming unequal variances). Thus the PDR, habituated by the 1-kHz narrowband noise, can be recovered by changing the center frequency to 2 kHz. Finally, as shown in Fig. 9b, which plots the size of the PDR evoked by only the oddball stimuli against trial number (magenta line and circles), the PDRs to the oddball stimuli of a different pitch also habituate, as we found for oddball stimuli that differed in SPL.

Taken together, these results demonstrate that the PDR can be habituated by the repeated presentation of a narrowband noise and at least partially recovered by altering the SPL or frequency. This recovery from habituation is specific to the oddball stimulus—presentations of the habituating stimulus following the oddball trial (Fig. 9, dotted lines) elicited PDRs that were no different from habituating trials before the oddball (Fig. 9, dashed lines). Thus, the recovery does not generalize to the habituating stimulus, but remains specific to the oddball.

## DISCUSSION

We describe an acoustically evoked pupillary dilation in adult human listeners, and show that the magnitude of the pupil’s response is stimulus level dependent. This suggests that the PDR method may serve as a way of assessing hearing in subjects for whom standard hearing tests cannot be used. A significant dilation was observed whenever listeners pressed the “yes” button, indicating detection, but not when they pressed the “no” button, suggesting that the dilation was not due to the intent to push a button or the motor act thereof. This result resembles earlier findings by Hakerem and Sutton (1966), where subjects were asked to report whether or not they detected a near-threshold visual stimulus, and found that pupil size was larger during trials when subjects signaled that a light was seen, compared to trials when subjects signaled that no light was seen. Furthermore, as in Fig. 2, voluntary detection in the Hakerem and Sutton study was signaled after pupil size was recorded, excluding the possibility that the act of pressing a button itself caused a pupil dilation (Richer et al. 1983). Note that the absence of the dilation response during catch trials shows that the dilation was due to the sound, and not due to other factors, such as decreased light level, or a reaction to the change in the cuing symbol on the monitor, both of which remained identical during sound and catch trials.

The dilation observed had a relatively short latency (≈ 0.25 s) and persisted for about 2 s at supra-threshold SPLs. As in the barn owl (Bala and Takahashi 2000), the adult human PDR was also found to scale in magnitude with the amplitude of the sound. When comparing thresholds obtained in simultaneous PDR/DRT trials, the PDR was somewhat more sensitive but more variable across multiple test sessions within individuals. Finally, we demonstrated that the PDR habituates with the repeated presentation of a narrowband noise-burst, but recovered in the oddball trials where the center frequency was altered or the SPL *decreased*.

Kahneman (2003) distinguished between an early, phasic pupillary dilations, corresponding to the Pavlovian orienting response and a later, sustained dilation. The later component, which is observed during the expenditure of cognitive effort has been used to study processes such as the recruitment of attentional resources, the updating of working memory (Einhäuser et al. 2008; Raisig et al. 2010; Hochmann and Papeo 2014; Unsworth and Robison 2015), and the effort expended during a listening task in normal hearing and cochlear-implant users (Koelewijn et al. 2014; Steel et al. 2015; Winn et al. 2015). By contrast, our study exploited the earlier orienting response evoked by changes in the auditory environment.

### Comparison of PDR and DRT

The PDR is a potential tool to assess auditory detection and discrimination in those who are not able to participate in hearing tests that require a voluntary response, such as the Hughson-Westlake procedure. Because we monitored the pupils as listeners performed the DRT, we were able to compare the two methods both across and within individual subjects to gain a preliminary estimate of how the voluntary (DRT) and involuntary response (PDR) compare. In individual listeners in which PDR and DRT thresholds were obtained *at the same time*, analysis showed that at four center frequencies tested, the PDR thresholds were statistically lower than those obtained with the DRT.

While the mean thresholds obtained with the two methods were similar, the variance of thresholds obtained with PDR was larger than those obtained with DRT (Fig. 6b–e). One possibility is that in the DRT, the neural mechanisms that evaluate the decision to press a button, may be more stringent than the mechanism leading to dilation, requiring a greater difference in the neural activity during catch and test trials before a subject presses the yes button. This would lead to more consistent thresholds across sessions. *By contrast, the PDR circuitry may evaluate the neural activity independently of the conscious decision process, and as a result, the thresholds for pressing the yes button and dilating the pupil may be different.* This view of the PDR as a process independent of the conscious decision is consistent with our finding that PDR magnitude scales with SPL, and is already rising at sound levels that appear to be below the voluntary detection threshold (e.g., Fig. 5a at 33 dB; Fig. 5b at 23 dBA).

Had they not been scarce, the false-alarm rate would have shed light on whether the dilation reflects a conscious or pre-conscious detection of the sound. Specifically, during a false alarm, a subject would indicate by button press that a sound was presented during a catch trial. If the PDR is observed in this case, it would suggest that the pupillary response reflects the subjects’ mistaken judgment that a sound had been presented. Alternatively, if the PDR is not observed, it would suggest that the PDR is evaluating the neural evidence independently of the process leading to a conscious decision. Further studies using paradigms designed to increase the incidence of false alarms, e.g., by the addition of masking noise, may lead to a clearer picture.

The fact that no voluntary response is needed for the PDR may make it more vulnerable to environmental noise that can diminish the difference between the neural activity evoked in test and catch trials. Since the sessions were conducted in a quiet office rather than a sound-isolating booth, the neural activity in the catch trials may have been boosted by environmental noise. A related potential reason is that the spatial resolution of the pupillary images afforded by the *Eye Link* system was relatively coarse. Small fluctuations in pupil size, due to the hippus or to external noise, will lead to a bigger proportional change if the number of pixels contained in the pupillary image is small.

As noted in the “INTRODUCTION,” our goal was to get an initial impression of how the PDR and DRT compare in sensitivity and repeatability, and not to show that the PDR, in its current state of development, is a suitable substitute for the voluntary task in a clinical setting. The latter requires more extensive testing. Still, it is encouraging that despite the potential differences in the internal motor machinery at work during the DRT and PDR, similar thresholds were obtained with the two approaches. To go beyond our preliminary assessment, we will first need to reduce the level of ambient acoustical noise and to image the pupil at a higher spatial resolution. For use in infants, the pupil will have to be tracked during unexpected head or eye movements. These efforts are currently under development (unpublished data, Bala and Takahashi).

### Habituation and Recovery of the PDR

A habituating-recovery paradigm can potentially be exploited as a reporting tool to examine sensory discrimination by habituating the PDR with one set of stimulus parameters and testing for recovery by altering one of the parameters, an approach we have previously used to determine auditory discrimination thresholds in barn owls (Bala and Takahashi 2000; Bala et al. 2003, 2007; Spitzer et al. 2003).

We observed that when a narrowband noise was presented repeatedly at a supra-threshold SPL, the PDR habituated rapidly such that the second trial was considerably smaller than the initial, novel presentation (Fig. 7). However, as in the owl (Bala and Takahashi 2000), even after the first trial, a diminished dilation remained (Fig. 7, *trials 2 to 30*), indicating that the sound had registered in the auditory system. During an oddball trial, the pupil dilated beyond the habituated response (Figs. 8, 9), suggesting that the difference between habituating and oddball stimuli had been detected.

Above, we showed that the PDR recovered when the center frequency or amplitude of the stimulus was altered. Note that the recovery is specific to the oddball: as shown in Fig. 9, responses during habituating trials that immediately followed the oddball (dotted black lines and upright triangles) were no different from habituating trials that immediately preceded the oddball (dashed blue line and inverted triangles; paired *t* test, *p =* 0.05). Thus, the recovery of the PDR magnitude observed during the oddball presentation remained restricted to the novel stimuli, and did not result in non-specific dishabituation. This frequency-specific nature of the habituation and recovery is consistent with the view that the PDR is based on the habituation of the activity in frequency-specific neurons. When the center frequency is shifted, the activity shifts to neurons tuned to another frequency that have yet to be habituated. A similar model was successful in linking the spatial-discrimination behavior of the owl to changes in activity of spatially selective neurons in the auditory space map of the owl’s inferior colliculus (Bala and Takahashi 2000; Bala et al. 2003, 2007). Further, the fact that the PDR habituates, and recovers in a stimulus-specific manner, confirms that the sound-evoked response we measured matches the previously described properties of the orienting response (Sokolov 1963; van Olst 1971; O’Gorman 1979), differing in significant ways from tasks that track longer-term effects due to cognitive effort (e.g., Koelewijn et al. 2012, 2014; Hartmann and Fischer 2014; Unsworth and Robison 2015).

The recovery observed by changing the SPL (maintaining the same center frequency) is particularly interesting. As shown above, a habituated PDR could be recovered by *decreasing* the SPL. This result is also an interesting contrast to our findings in the Audiometry section (Experiment 1) that sounds at higher SPL elicited larger PDR responses. Thus, in the habituation-recovery context, it is not the level of the sound that matters, but its novelty, suggesting that PDR recovery is due to the detection of a change in the *absolute value* of the difference in neural activity generated by habituating and oddball trials.

The difference in the PDR to habituating and oddball stimuli may be used to evaluate discrimination thresholds in other perceptions. For example, the PDR habituation/recovery may be used to assess the ability to discriminate between phonemes. While standard audiometry indicates the detectability of narrowband sounds at various SPLs, speech sounds, such as /*ba*/ and /*pa*/, differ not in the average power spectra but by *changes* in the spectrum *over time*. Phoneme discrimination in infants is of obvious clinical interest, but measuring discrimination rigorously currently requires multiple personnel and a lengthy period of time for training the infant to respond (Olsho et al. 1987; Hicks et al. 2000). The PDR, which only requires video-monitoring of the eyes, may enable the routine testing of speech-sound discrimination in infants, which, in turn, may indicate whether or not a child’s speech development is progressing normally.

However, some optimization is required before the PDR can be used to assess sensory discrimination in a research or clinical setting. For example, can the effects of habituation be reduced? Our data shows that the pupil response habituates to repetition of not just the habituating, but also the oddball stimuli (Fig. 9). If this always held true, the utility of the method would be vastly decreased. However, the effects of habituation can be reduced.

Separating repeated stimuli in time and introducing other sounds in intervening trials are known to diminish the rate of habituation (Coombs 1938; Geer 1966; Simon 1976). Our data show the same effect: habituation to repeats of the habituating stimulus (Fig. 7) produces a rapid decrease in stimulus magnitude during after only two or three repeats, followed by complete habituation after about 30 trials (Fig. 7). However, the second and third iterations of the oddball stimulus, which are separated by a much longer interstimulus interval (ISI) of 160 s for oddball vs 8 s for the habituating stimulus, show a smaller reduction in response magnitude (Fig. 9, red and magenta lines). Thus, our data confirm earlier observations, where separating successive repetitions of a given sound stimulus in time, and adding more intervening trials, reduces the effects of habituation.

**Fig. 10:**
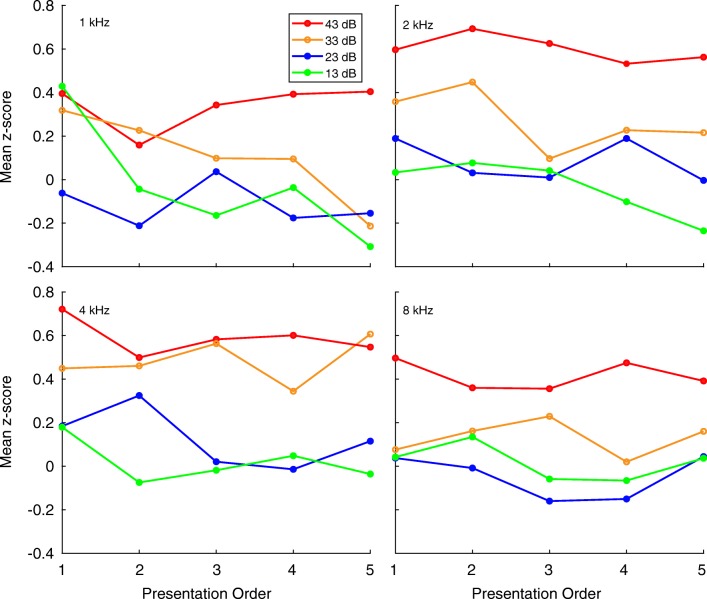
Trial order effect at near-threshold SPL. PDR sizes (ordinate) evoked by five repetitions (abscissa) of a 1-, 2-, 4-, and 8-kHz gammatone, each at one of four SPLs (13, 23, 33, 43 dBA). Each iteration of a sound-level combination was separated from the next one by ≈ 240 s. There is no systematic, statistically significant change in response magnitude (*t* test, *p =* 0.05) to later presentations of the sounds at any frequency, in contrast to the evidence of rapid habituation shown in Figs. 7 and 9

In addition to ISI, the amplitude of the habituating stimulus also affects habituation. In the owl, the PDR habituated rapidly at SPLs well above its threshold. Nearer to threshold, however, it did not habituate (Bala and Takahashi 2000), which is consistent with the observations that human heart rate and galvanic skin responses were resistant to habituation at the lowest SPLs tested (Jackson 1974). Our data are consistent with these observations. While stimuli presented at higher levels (53 dBA) during habituation and oddball experiments showed significant habituation, we saw none when sound levels were within 20 dB of detection threshold during Experiment 1. This is shown in Fig. 10, which plots the *z*-score of pupil sizes (re: catch trials without sound) against the order in which the gammatones were presented (horizontal axis) at each of the four levels used. In these data, there is no statistically significant change in PDR magnitude with the order in which gammatones were presented. Thus, the rate and amplitude with which the habituating stimuli are presented, when optimized, can reduce the effects of habituation, and increase the efficiency of the PDR.

The present study provides evidence suggesting that upon determining the optimal presentation rate and SPLs of the test stimuli, the PDR method may be used to explore and evaluate human auditory detection and discrimination. With some development (Bala et al. 2018), this method may prove especially valuable in subjects who are unable to follow instructions (Winn et al. 2015) or give a reliable response.
